# If You Build It, Will They Come?: Getting the Word out About Gerontology Accreditation

**DOI:** 10.1093/geroni/igab046.160

**Published:** 2021-12-17

**Authors:** M Aaron Guest, Phillip Randall

**Affiliations:** 1 Arizona State University, Phoenix, Arizona, United States; 2 The Thorndike Group, Minneapolis, Minnesota, United States

## Abstract

The development of AGEC introduced a new facet to gerontological education: accreditation. The presence of such a new organization requires continuing marketing and education. Throughout its first three years, AGEC has continually worked to provide information and to differentiate between accreditation and credentialing for the broader community. Through informal and formal feedback processes, including focus groups and interviews, AGEC has refined its message and delivery. While schools and departments of Gerontology remain the decision-makers behind seeking accreditation, students have become one of the largest drivers and constituencies AGEC engages as they seek clarification on and the availability of accredited programs of Gerontology. Prospective students, many coming from the health sciences, see the value of accreditation. Furthermore, emerging and international programs see accreditation as an opportunity to engage the field. There is an opportunity to further refine the messages around accreditation and differentiate among the organizations active in gerontological education.

